# Editorial: Interactions Between Small Molecule Ligands and Target Enzymes

**DOI:** 10.3389/fmolb.2021.649450

**Published:** 2021-03-05

**Authors:** Sung-Kun Kim, Ki Duk Park, Dong-Woo Lee

**Affiliations:** ^1^ Department of Natural Sciences, Northeastern State University, Broken Arrow, OK, United States; ^2^ Brain Science Institute, Korea Institute of Science and Technology, Seoul, South Korea; ^3^ Department of Biotechnology, Yonsei University, Seoul, South Korea

**Keywords:** enzyme, ligand, interactions, drug discovery, molecular dynamics

The detail of the interactions between enzymes and ligands is a prerequisite for understanding cellular processes and developing novel therapeutic agents and chemical drugs. Although enzyme-ligand interactions occur via the complex formation driven by thermodynamics, the rate of complex formation and breakdown should also be considered on the reaction coordinate using kinetic parameters and thermodynamic stability. To gain insight into the molecular basis of enzyme-ligand interactions at the atomistic level, three-dimensional structural analysis of enzyme-ligand complexes is necessary. Nevertheless, it is noted that the protein-ligand interactions initiate an important conformational change and the binding states vary over the reaction period, representing a major challenge for the development of refined and optimized poses from the initial docking state. Accordingly, computational approaches have been conducted to advance the understanding of enzyme-ligand interactions. Recent advances in molecular docking and dynamic algorithms have provided more reliable and accurate illustrations of the enzyme-ligand complex, leading to the development of new regulatory ligands for target enzymes or protein engineering for better interactions.

In this research topic, we introduce the research articles and reviews tackling the interactions between enzyme and ligand using a wide variety of state-of-the-art techniques. We believe that the current, updated ideas and/or strategies to identify and develop new ligands for target enzymes can enhance the current understanding of structural flexibility or mutational effects on the ligand and its target, thus making an essential contribution to the fields of drug design and biotechnology.

The computational approaches are getting more critical to detailing the information of interactions between ligands and enzymes. The accuracy is substantially improved. Molecular dynamics simulation is the spearhead of the computational approaches to provide structural information. Adams et al. explained how the molecular dynamics simulations provide insight into antibiotic interactions using the enzyme l,l-diaminopimelate aminotransferase with the potential inhibitors. This study supports the great potential scope of the application of molecular dynamics to biological molecules to infer the function, form, and effect of interactions in situations where the crystal structure may not be known. Another example of a computational approach is an article published by Shaker et al. The authors attempted to design potent inhibitory lead molecules against *Pseudomonas aeruginosa* using an *in silico* ligand-based drug design. Subsequently, the researchers conducted a structure-based virtual screening with a library of potential inhibitors for anthranilate-CoA ligase, which is involved in the quorum signaling system of *P. aeruginosa*. The molecular dynamic simulation further validated resulting compounds in identifying improved inhibitors. Such a computational approach might provide reasonably acceptable drug candidates against the resistant phenotypes of opportunistic infectious pathogens.

In this research topic series, two review articles described drug discovery. Hwang et al. introduces the structure and mechanism of modular PKS and NRPS, and includes examples of their repurposes. In addition, the authors discussed the tools of the design-build-test-learning cycle of synthetic biology and the future perspective of repurposing strategies. Vellanki et al. reviewed the recent effort in the discovery of novel non-immunosuppressive antifungal drugs against fungal pathogens. This review highlights the interactions of immunosuppressive drugs FK506 and rapamycin with immunophilin FKBP12 in targeting calcineurin and TOR kinase in human and fungal pathogens. Such a detailed inspection of ligand-bound protein structures will provide a useful guide to facilitate the development of new small molecules with antifungal activity.

Some research articles presented current issues we are facing. For example, extended-spectrum β-lactamases (ESBLs) are a serious threat to human health due to their enhanced hydrolytic activity against third-generation cephalosporins. Cao et al. described the structural mechanism underlying the hydrolysis of third-generation cephalosporins using non-catalytic-region (NCR)-ESBLs. Also, melanin, an important molecules in humans, plays a crucial role in protecting the skin from the harmful effects of ultraviolet radiation, but the abnormal accumulation of melanin causes skin pigmentary disorders. Kim et al. proposed a molecular mechanism for how 7,3′,3′-trihydroxyisoflavone (7,3′,4′-THIF) attenuates α-MSH-induced melanogenesis.

Interactions between ligands and proteins reveals many important biological and biochemical aspects. For example, protein-ligand binding structures enabled us to identify the functional role of a thermostable primordial protein. La et al. described the functional characterization of a gene encoding a putative keratin-degrading β-aspartyl peptidase. Determination of its co-crystal structure with the substrate analog together with *in vivo* complementation experiments suggest that the viability of hyperthermophiles under stressful conditions may rely on the activity of BAP within cellular protein repair systems. Additionally, Kwon et al. describes the development of a fluorescent sensing system for monitoring enzyme activity in living cells. Specifically, the authors designed a DmpR-based genetic circuit relying on para-nitrophenol and alanine as regulators interacting with tyrosine phenol-lyase, allowing for straightforward control of its output signal without additional genetic modification. The developed genetic circuit system can be a useful tool for the accurate and rapid analysis of targeted enzyme activity in living cells.

The articles published here discussed a broad range of state-of-the-art computational and experimental studies, or a combination thereof, to understand the interactions between small ligands and their target biomolecules. We believe these articles help to emphasize the impact of the investigated processes in biomedicine and applications of enzyme-ligand interactions in biotechnology and other related science fields.

